# Incidence and risk factors of venous thromboembolism (VTD) in patients with amyloidosis

**DOI:** 10.1186/1477-7800-2-17

**Published:** 2005-09-02

**Authors:** Gordan Srkalovic, Marte G Cameron, Steven R Deitcher, Kandice Kattke-Marchant, Mohamad A Hussein

**Affiliations:** 1 Sparrow Regional Cancer Center, Lansing, Michigan, USA; 2 Helgelandssykehuset, Avdeling Sandnessjøen, Sandnessjøen, Norway; 3 Nuvelo Inc, Sunnyvale, California, USA; 4 Department of Pathology, Cleveland Clinic Foundation, Cleveland, Ohio, USA; 5 Cleveland Clinic Myeloma Research Program, Cleveland Clinic Foundation, Cleveland, Ohio, USA

## Abstract

**Background:**

Coagulation problems in amyloidosis are historically associated with bleeding tendencies (mostly Factor X abnormalities). Increased clotting was observed in isolated cases diagnosed with low-grade disseminated intravascular coagulation (DIC). Problem of venous thromboembolic disaease (VTD) in amyloidosis was not systematically investigated.

**Methods:**

We evaluated frequency of VTD and risk factors for VTD in 56 consecutive amyloidosis patients with a documented disease evaluated and followed up at our Center from 1991–2001. Data was collected in 5 categories: (a) demographics, (b) disease and treatment, (c) thrombosis case information, (d) major risk factors for thrombosis and (e) baseline laboratory data. Univariable correlates of VTD were assessed using Kaplan-Meier analysis and Cox proportional hazards analysis.

**Results:**

Mean age of the patients was 67 (years range 21 – 83). Male/female percentage ratio was 70/30. 29 % of the patients had high creatinine level (> 1.4 mg/dl). Personal or family history of VTD was recorded in 2 and 0 % of patients, respectively. Known hypercoagulable state was present in 1 patient (2%). 8 % of patients were smokers. Of 56 patients, 6 developed VTD (11%). Median time from diagnosis of amyloidosis to VTD was 12.5 month (range 1–107). Treatment was given within a median of 1 month (range 0–4) from the development of thrombosis. Only sites of VTD were lower extremities. No cases were associated with I.V. line. 1 case (17 %) was identified postoperatively. We identified several univariable correlates of VTD in amyloid patients, including greater age at diagnosis (HR-2.99, P = .041), personal history of DVT (HR-47.7, P = .006) and immobility (HR-11.78, P = .006). Presence of circulating serum M-protein had protective role in our analysis (HR-.08, P = .031). There was no correlation with the type of treatment patients were receiving.

**Conclusion:**

Risk for thromboembolic diseases in patients with amyloidosis is similar to one previously described for multiple myeloma. Additional studies with higher number of thromboembolic events could help to further elucidate risk factors for VTD in this population of patients.

## Background

amyloidosis is characterized by organ deposition of fibrillar substances of different types that have a homogeneous eosinophilic appearance on light microscopy [1,2]. The diagnosis is confirmed by demonstration of an apple-green birefringence under polarized light of specimens stained with Congo red. Primary amyloidosis (AL) should be considered in any patient who has a monoclonal protein in the serum or urine and refractory congestive heart failure, nephrotic syndrome, sensorimotor peripheral neuropathy, carpal tunnel syndrome, orthostatic hypotension, or steatorrhea. At diagnosis, approximately one-third have nephrotic syndrome, one-fifth have carpal tunnel syndrome, one-fourth have congestive heart failure, and about 15% have peripheral neuropathy [1,2].

Bleeding problems due to vascular deposition of amyloid resulting in vascular fragility, deficiencies of clotting factors (particularly Factor X) or enhanced fibrinolytic activity often complicate this disease [3]. Acquired deficiency of Factor X is the most common coagulation factor deficiency identified in patients with amyloidosis [4]. Thrombosis, although less common, also can occur. Some case reports described thromboembolic complications of amyloidosis [5,6]. Association of nephrotic syndrome, well-documented complication of systemic amyloidosis, and renal vein thrombosis are well documented [7,8]. Cardiac amyloidosis produces cases of atrial electromechanical dissociation with resulting atrial arrhythmias and formation of atrial clots [9]. Various pathogenic factors have been proposed to explain abnormal hemostasis in patients with amyloidosis. Recently, impairment of the thrombin-antithrombin pathway, in association with the low antithrombin biological activity was recognized to have possible pathogenic role in hypercoagulability of amyloidosis patients [10].

However, the problem of venous thromboembolic disease (VTD) in this disease was not systematically investigated. We evaluated the frequency of VTD and risk factors for VTD in 56 consecutive amyloidosis patients with a documented disease evaluated and followed up at our Center from 1991–2001.

## Methods

We reviewed complete medical records of all patients with amyloidosis who were prospectively entered in the database. Fifty-six patients were identified. They were evaluated and followed up by staff oncologists in the Department of Hematology and Oncology, Myeloma Research Program at the Cleveland Clinic Foundation in the period 1991–2001.

The Cleveland Clinic Foundation (CCF) is an urban tertiary referral center and a teaching hospital. Our proposal was submitted to the CCF Institutional Review Board, and we were granted approval, based on an expedited review. The requirement for obtaining informed consent was waived based on the study designs (standard medical record based review) and patient anonymity. The study did not require any third party funding.

The patients' computerized inpatient and outpatient medical records were initially reviewed by one of the co-investigators (CMG). Additional review was done independently by a second co-investigator (SG). If electronic data were incomplete, the hard copies of charts were also reviewed. When necessary, missing information was sought from referring providers. However, in a number of cases, this was unavailable.

All cases of VTD of extremities were diagnosed by Duplex ultrasound. Term immobility was used for patients who were hospitalized or bed bound for more than 7 days at the time of VTD diagnosis.

Personal history of VTD was defined as a history of documented thromboembolism prior to the diagnosis of PCD. Patients were screened for a hypercoagulable state only when they developed what appeared to be a vascular event that needed further investigation.

Data was collected in 5 categories: (a) demographics, (b) disease and treatment, (c) thrombosis case information, (d) major risk factors for thrombosis and (e) baseline laboratory data. Data were manually recorded onto researcher-developed data collection forms and subsequently entered into a computerized database.

### Statistical analysis

Categorical variables are summarized as frequencies and percentages and continuous variables as the mean, standard deviation, median, and range. Time until development of a VTD was estimated using the Kaplan-Meier method. Follow-up was calculated from the date of diagnosis to the date of either the first VTD or the date of final follow-up visit. Cox proportional hazards analysis was used to identify univariable correlates of thromboembolic event. If Cox analysis could not be done due to lack of variability, then the log-rank test was performed instead.

There were 8 possible risk factors for VTD: personal and family history of VTD, known hypercoagulable state, non-plasma cells related concomitant neoplasm's, immobility, estrogen treatment, smoking, and use of indwelling catheters at any time from initial diagnosis.

All statistical analyses were two-sided; P < 0.05 was used to indicate statistical significance. Analyses were performed using SAS^® ^software, version 6.12.

## Results

### Patients' characteristics

Data was available on 56 patients (Table 1.). 50 (89%) had primary amyloidosis (AL), 1 (2%) had kappa light chain disease, and in 5 (9%) patients type could not be identified. One patient had both AL and familial amyloidosis. 37 (66%) had confirmed cardiac involvement by biopsy or alternatively, typical findings on Echocardiogram. Confirmed (biopsy) renal involvement was found in 18 (32%) of patients. Localized amyloidosis was identified in 6 (11%) of patients. Two cases were pulmonary, 2 gastrointestinal, and one case was localized to skin and genitourinary tract, respectively. 39 (70%) were men and 17 (30%) were women. Average age was 67 years (range 21 – 83). Of the heavy chain types found in patients, 75% were IgG, 17% IgA, 1% IgM. 44/56 (78.5%) of patients did not have heavy chain M-protein in the serum.

**Table 1:** Clinical characteristics of amyloidosis patients at presentation (N = 56)

**Characteristics**	
**Male/Female**	39/17

**Age (years)**	
Median	67
Range	(21–83)

**Serum M-Protein (g/L)^1^**	(N = 18)
Mean	0.5
Median	0.2
Range	(0–4.0)

**24-hour Urine Protein (g)^1^**	(N = 47)
Mean	3.2
Median	0.4
Range	(0–26.9)

BMPC* (%)^2^	(N = 53)
Mean	8
Median	5
Range	(0–50)

**B_2 _Microglobulin (mg/dl)^1^**	(N = 47)
Mean	3.4
Median	2.7
Range	(0.3–18.4)

**Serum albumin (g/L)^1^**	(N = 53)
Mean	3.4
Median	3.6
Range	(1.0–5.4)

**Immunoglobulin type Heavy Chain**	(N = 12)
IgA (%)	2 (17)
IgG (%)	9 (75)
IgM (%)	1 (8)
Unknown	0
**Light Chain**	(N = 25)
λ	18 (72)
κ	7 (28)

**History of VTD Personal**	
Yes (%)	1 (2)
No (%)	53 (94)
Unknown (%)	2 (4)
	
**Family**	
No (%)	52 (93)
Unknown (%)	4 (7)

^1 ^CCF Laboratory reference range, normal values:

Serum M-protein: 0 g/dl

Urine Protein: 0–0.15 g/24°

β_2 _Macroglobulin: 0.3–1.9 mg/dl

Serum Albumin: 3.5–5.0 g/L

^2 ^Bone Marrow Plasma Cells

The light chain was lambda (λ) in 18 (32% of all patients) and kappa (κ) in 7 patients (13%). The remainder did not have identifiable light chain (31, 55%). All patients had amyloid deposits by Congo Red stain. The bone marrow biopsy results were available in 53 patients (95%) at the time of diagnosis. The median percentage of bone marrow occupied by plasma cells was 5% (range 0.0 – 50%). Serum creatinine level was elevated in 29% of all patients. Albumin was low (< 3.5 gm/dl) in 45% patients overall. Beta-2-microglobulin was high (>1.9) in the majority of patients (70%). Bone survey was negative for lytic lesions in all cases.

Out of 56 patients with amyloidosis, 1 (2%) patients had personal and no one had family history of thromboembolism. 1/56 were diagnosed with hypercoagulable state (antithrombin deficiency). Patients were treated with multiple regimens that included: steroids (36%), α-interferon (23%), vincristine (16%), liposomal doxorubicin (16%), melphalan (9%), thalidomide (2%) and rituximab (2%).

The five different drug combinations used in the treatment of amyloid patients are presented in Table 2. No patients were treated with high-dose chemotherapy with peripheral stem cell support (HDC-PSC).

**Table 2:** Combination chemotherapies used in the treatment of amyloidosis

**Type of Treatment**	**N**	**%**
HD steroids^1^	20	36
DVd^2^	9	16
MP^3^	5	8
CP^4^	1	2
TD^5^	1	2

N – Number of patients

% – Percentage of patients treated with particular regimen

^1 ^High-dose Dexamethasone

^2 ^Doxil^®^, Vincristine, Dexamethasone

^3 ^Melphalan, Prednisone

^4 ^Cytoxan, Prednisone

^5 ^Thalidomide, Dexamethasone

### Patients with VTD

Cases of thromboembolism were diagnosed by Duplex ultrasound. There was no evidence of pulmonary embolism or atrial clots in this group of patients. Of 56 patients reported in this paper 6 (11%) had one or more episodes of symptomatic, objectively documented VTD after the diagnosis of amyloidosis (Table 3). All cases were of lower extremities. The time from diagnosis to development of VTD was 12.5 months (range 1–107). Median time from last treatment to VTD event was 1 month (0–4). Out of 6 patients with VTD 17% (1 case) had a personal history of thromboembolism. No patients with VTD had a history of hypercoagulable state, smoking, or oral estrogen use. Four (67%) patients with VTD had prolonged immobility and 1 (17%) had indwelling catheter at some point during the disease. One patient (17%) developed VTD within 6 weeks from surgery.

**Table 3:** Clinical characteristics of amyloidosis (N = 6) patients with VTD

**Characteristics**	
**Male/Female**	4/2

**History of VTD**	
**Personal**	
Yes	1
No	5
**Family**	
No	6
**Serum M-Protein^1^**	
None	3
Yes	2
Unknown	1

**24-hour Urine Protein^1^**	
<0.15 gr	3
>0.15 gr	2
Unknown	1

**BMPC^2^**	
≤ 5%	3
> 5%	1
Unknown	2

**B_2 _Microglobulin^1^**	
< 1.9 mg/L	1
> 1.9 mg/L	4
Unknown	1

**Serum albumin^1^**	
< 3.5 g/L	3
3.5 – 5.0 g/L	3

**Immunoglobulin type**	
None	4
IgG	1
IgM	1
	
None	4
κ	2

**WBC count**	
4 – 11 × 10^6^/L	6

**Platelet count**	
150 – 400 × 10^6^/L	6

^1^CCF Laboratory reference range, normal values:

Serum M-protein: 0 g/dl

Urine Protein: 0–0.15 g/24°

β_2 _Macroglobulin: 0.3–1.9 g/dl

Serum Albumin: 3.5–5.0 g/L

^2 ^Bone Marrow Plasma Cells

Univariable correlates of VTD in amyloidosis patients are presented in Table 4. A large number of factors were entered into the model including the following: gender, age at diagnosis, immunoglobulin type, personal history of VTD, hypercoagulable state, non-plasma cell related concomitant neoplasms, smoking, immobility, indwelling catheters, estrogen treatment, percentage of plasma cells in the bone marrow biopsy, creatinine level, corrected serum calcium level, serum albumin, white blood cells and platelet count, hemoglobin level, β 2-microglobulin, serum M protein level, 24 hour urine protein level, as well as individual agents and combination of chemotherapeutic agents.

**Table 4:** Identifying univariable correlates of VTD in amyloidosis (N-56)

**Variable (Reference)**			
	**HR^2^**	**95% CI^2^**	**P^1^**

Age at Diagnosis (per 10 year increase)	2.99	1.05–8.56	0.0411
Personal History of VTD (Yes/No)	47.7	2.98–766	0.0061
Immobile (Yes/No)	11.78	2.02–68.8	0.0061
Serum M-protein			
Per 1 mg/dl increase	0.43	0.05–3.70	0.45
Normal/Abnormal	12.5	1.25–100	0.0311

^1 ^significant (P < 0.05)

^2 ^HR – Hazard Ratio; CI – Confidence Interval

Among these, personal history of VTD (HR-47.7; P = .006, CI-2.98–766), immobility (HR-11.78; P = .006, CI-2.02–68.8), absence of measurable M-protein (HR-12.5; P = .031, CI-1.25–100), and age at diagnosis (HR-2.99, P = .041, CI-1.05–8.56) were identified as univariable correlates of VTD in amyloidosis patients (Table 4.) Multivariable analysis could not be done due to low number of events.

## Discussion

This study shows increased incidence of VTD (11% at 10 years review) in amyloidosis patients as compared to the general population (0.1% per person/per year) [11]. This is similar to frequency of VTD found in multiple myeloma (MM) (10%) [12], and slightly higher than in patients with monoclonal gammopathy with undetermined significance (MGUS) (7.5%) [12]. Previously, only case reports of thromboembolism in patients with amyloidosis were presented in the literature [5,6,9,13].

Studies presented herein have significant limitations. It is highly dependent on the quality of historical data. Absence of a potential risk factor or patient characteristics in the medical records is assumed to mean that the factor is absent. The statistical analysis is limited by the fact that several of the variables that were considered as correlates of VTD were not baseline characteristics. Ideally, these variables would be analyzed as time-dependent covariates. However, timing of these variables was not known. It is only known that they occurred before development of the event in patients with VTD or before the last follow-up among patients who did not develop VTD yet.

Results of the study presented herein point to strong correlation between some of the established risk factors for VTD (personal history and immobility) and thromboembolic events in patients with amyloidosis. Other well-known VTD risk factors (estrogen use, concomitant solid malignancies, and use of indwelling catheters) did not seem to be of importance in our patients.

Data about family history of VTD is missing in more than 10% of patients. Additionally, existing data seem to be inadequate. From available records it seems that none of the 56 patients had a family history of VTD. Although this could be due to the low number of patients a more probable explanation is poor history taking. Therefore; family history was not included in univariable analysis. Types of treatment (individual drugs or chemotherapy combinations) do not correlate with VTD. It is important to mention that none of the patients included in this study had treatment with high dose chemotherapy and stem cell support.

One of the univariable correlates of VTD identified in our group of amyloidosis patients is increased age. Risk of VTD in these patients is increasing 3 times with every 10-year increase in age. A number of studies support an association between increasing age and a higher incidence of venous thromboembolism [14-16]. Patients over age 40 are at a significantly increased risk compared with younger patients [17]. Risk continues to increase with increasing age, approximately doubling with each decade after age of 40 [18].

It seems that in our amyloidosis patients, VTD age risk is in excess of that seen in general population. It does not seem that this increased risk correlates with more aggressive disease, since age was not found to be an important prognostic factor for survival in primary systemic amyloidosis in multiple studies [19-21]. However, other types, such as senile with normal transthyretin (ATTR), amyloidosis associated with Alzheimer's disease (Aβ), and islet amyloid polypeptide deposits (AIAPP) are very strongly associated with both old age and the aging process.

Another significant risk factor of VTD in our patients was absence of circulating monoclonal protein. This is a characteristic for types of disease other than primary amyloidosis (AL) including secondary (AA), familial with genetically variant transthyretin (ATTR), amyloidosis associated with dialysis (Aβ_2_M), as well as Aβ and AIAPP forms. Again, these results point to possible increase incidence of VTD in the forms other than primary.

Human serum amyloid A (SAA) is a precursor protein in inflammation-associated secondary amyloidosis (AA). It could interfere with the coagulation process through interaction with heparin/heparan sulfate at the specific binding site (C-terminal residue 78 to 104) [22]. SAA also seems to play a role in atherogenesis, as well as in thrombus formation at the vascular injury site [23,24]. Secondary amyloidosis (AA) is manifested in patients with underlying chronic inflammation (rheumatoid arthritis, ankylosing spondylitis, inflammatory bowel disease, tuberculosis, osteomyelitis, Familial Mediterranean Fever) [25-27]. It is driven by different cytokines, including tumor necrosis factor (TNF-α), interleukin-6 (IL-6), and interleukin-1 (IL-1) [28]. IL-6 was found to promote coagulation without affecting fibrinolysis [29]. It activates coagulation cascade through tissue factor stimulation and increased transcription of factor VIII, up regulates transcription of fibrinogen, increases von Willebrand's factor, and decreases protein S [29]. Is there any correlation between SAA or any other circulating non-monoclonal protein precursors of amyloid and VTD will have to be elucidated in future studies. It is still uncertain if proposed increased incidence of thromboembolism is the result of the very process of deposition of the abnormally folded protein outside cells, consequential organ damage, or alternatively, underlying processes leading to amyloid deposits. However, it does not seem to be related to the type of treatment used in this disease. This is in accordance with our own findings in the patients with multiple myeloma and MGUS [12].

## Conclusion

Our analysis confirmed that well-known risk factors for thromboembolism including personal history of VTD and immobilization could represent confounding factors in amyloidosis patients, too. However, additional correlates have to be considered, including type of disease and patients' age.

In our opinion, these patients need to be closely monitored for development of VTD. Standard thromboembolic prophylaxis should be used with extreme caution, considering bleeding tendency in the patients with amyloidosis.

In our opinion development of prospective studies dealing with venous thromboembolism in amyloidosis patients is of utmost importance. We will need to define pathophysiological mechanisms responsible in order to employ the most effective prophylactic measures.

## Competing interests

The author(s) declare they have no competing interest.

## Authors' contributions

GS have made significant contribution to conception and design of the study, analysis and interpretation of the data and drafting the manuscript

MGC have been involved in collection, analysis, interpretation of the data and critical revision of the manuscript

SRD have been involved in drafting and revising the manuscript

KKM have been involved in revising manuscript for important intellectual content

MAH have made significant contribution to concept and design of the study, revision of the manuscript and have given final approval of the version to be published.

All authors read and approved manuscript
